# Germicides and Antiseptics in Dentistry

**Published:** 1898-08

**Authors:** C. Bunting Colson

**Affiliations:** Charleston, S. C.


ARTICLE IX.

### GERMICIDES AND ANTISEPTICS IN DENTISTRY.

C. BUNTING COLSON, M. D., D, D. S., CHARLESTON, S. C.

Awarded First Prize ($200) in "Borolyptol" Prize Competition.

I take the liberty to plan this essay on the classification of the conditions indicating the subject, instead of classifying the drugs, believing in this manner I can more definitely present the subject.

The following is a list of pathological conditions, or otherwise, which includes nearly all the conditions that germicides and antiseptics are indicated in dental practice.

1st. Normal; Prophylaxy and Accidental Lesions.

2nd. Stomatocase, in three divisions: 1st, Aphthæ; 2nd, Thrush; Stomasitica; 3rd, Stomatitis Gangrenosa.

3d. Calcic Inflammation, Uilitis and Gingivitis.

4. Pragedenic Pericementitis.

5th. Intra-Osseous Suppuration.

6th. Dental Canal and Pulp Antiseptics, and Dental Canal Sepsis.

7. Disinfecting Instruments, etc.

1st. Prophylaxy—In a healthy mouth, no therapeutical agent is indicated; but there is a fast growing popular demand for a mouth lotion of an antiseptic and germicidal order to prevent disease and caries within the mouth. I am much opposed to giving any of the mouth lotions that are used in diseased conditions for *constant use* in a healthy mouth, as all, that is, all that have any therapeutic value are either stimulating, astringent, or alkaline, and will by long and constant use do injury. This is not a presumption, but a fact, noted from close observation. Cleanliness is the only prophylactic needed in health. I do not know of a single therapeutic agent or compound that I can advise *for continuous use*, but water and the intelligent use of the brush, silk or floss flag.

In accidental lesions with healthy surroundings I am still of the opinion that they are best left alone, unless symptoms of parasitical complications are evident, then treatment will be as mentioned under that division.

2nd. Stomatocace: 1st—Div.-Aphthæ. The several forms of aphthous stomatitis and its etiology cannot be given here, which is systemic and eruptive, breaking out in apparently perfectly healthy mucous tissues. We can not cure the disease by local treatment, but we can give much relief to our suffering patients and prevent the still more painful condition of parasitical complication. What antiseptic treatment or agent is giving the best results in the general treatment of these diseases? In my experience the salts of the basal element boron stand first. Boracic acid and the sodium biborate known as borax, when used with the proper media and strength, gives the best results in *preventing parasitical complications*, and act best as a specific to the lesions, as the boric acid seems to have a most gratifying therapeutical effect on the small excretory glands and follicles of the animal system. It is an old and ancient remedy, but gives the most gratifying results to-day as when the ancient mothers of the days of Herodotus placed it in the mouth of her aphthous cachectic baby, or rather in the mouth of her parasitical stomatical baby. Giving good results when indicated in young and old, and the mouth lotion that has it for its prime therapeutical agent may be depended on to give good results.

I will here include the 2nd division of stomatic diseases and treat as one in the further discussion on the antiseptic treatment of these lesions.

Division 2nd. Thrush or Stomatitis Parasitica—This disease, like the 1st division above, seems to inflict their presence during some abnormal condition of the system when it is poorly nourished, and the circulation imperfect, but in this disease it is positively a specific parasite that causes the painful lesions. As stated, that boric acid and biborate of sodium stand first among therapeutic agents in controlling and preventing the disease. It is used with most excellent results in the following compound mouth lotion, and by an experience of many years I can find no better form :

℞—For 5-ounce solution :

| | |
|---|---|
| Boric Acid, - - | 2 drams |
| Sodium Biborate, - - | 2 drams |
| Eucalyptus, - - | ½ dram (=½ ʒ) |
| Glycerine, - - - | 4 drams. |

Mix cold. Shake well before using.

Perfumed and made pleasant to Q. S. ad. cinnamon water 5 ounces, to which is added 10 drops of the oil of gaultheria and one grain of saccharine.

Now for a few words on the above and its use. It being an over-saturated solution, the powdered boric acid and the borax will remain largely undissolved in the solution, and I purposely have it so. The ordinary effect of aqueous mouth lotions remain in the mouth so short a time and particularly in stomatitic mouths that have an unusual salivary flow, all traces of the antiseptic agent is soon washed away. I have my lotion doubly charged with these floating powders that will lodge about the mouth for some time and give the continuous effect. Glycerine also assists it to remain in the mouth, and proves a very happy medium in assisting the boric salts. The official form of boro glyceride is used by some. I prefer the above as positively giving better results by its adaptability and longer therapeutical effect. So far this is very good treatment to control and prevent the increase of the disease, but the dentist who depends on the antiseptic and astringent mouth lotion alone, and wishes to give rapid relief to his patient, will get slow results, and for the rapid cure of these lesions a more positive treatment is indicated. It is a fact that in all the pathological conditions of the mouth, be the lesions eruptive or caused by organisms, the best results for the cure and relief is the positive treatment direct to the lesion by some powerful escharotic or asiringent antiseptic. For instance, in the eruptive ulcers of aphthæ, the ulcer should be washed off, or out if deep, with the three per cent. hydrogen dioxid, and the surface carefully touched over with either nitrate of silver or a tri. chlo. acetic acid crystal, or a twenty per cent. solution of sulphuric acid also gives excellent results. If done properly and the mouth kept clean antiseptically, the lesions immediately heal and lose the extreme painful state that they so often have. With the parasitical thrush lesions, this class, the treatment should vary according to the conditions. If it is only one or two places affected, wash the lesion off with strong bichloride of mercury solution, about 1 in 100, using a pledget of lint wet in the solution, then touch the surface over with the tri. chlo. acetic acid or nitrate of silver. But if the parasitical lesion has spread over a large surface, use the bichloride about 1 in 200 and wipe the surface over carefully. If any pus shows itself evident, by all means use the hydrogen dioxid in the same manner, and then apply a twelve per cent. solution of tri. chlo. acetic acid.

The pain of these applications soon passes away, and your patient is soon very comfortable and the cure rapid. It is always well to give the patient a good antiseptic mouth lotion of a quality that will last in the mouth, not too generally stimulating to the mucous membrane, as a very stimulating lotion, alcohol, and even some of the most generally used mouth lotions when used strong will bring on a state of very sore mouth and lips of aphthæ and Herpes vesicles. The lotion given should be used constantly during the course of the disease, not three or four times a day, but frequently to keep the influence of the antiseptic and germicide action constant.

A few words before passing the subject of antiseptic mouth washes, which I wish to impress as the most important idea expressed in this essay. It is very necessary to cleanse the mouth of all the viscid mucus that clings to the teeth and gums and about the mouth before using a mouth lotion to get its therapeutical action. The mucus is a most excellent culture media in the mouth for the many micro-organisms that infect the mouth and the general system. How shall we cleanse it or remove it? It is thought that germicide lotions will cleanse and sterilize, but it does not. This is also not an assumption, but an assertion made after numerous practical experiments and tests with all the advantages of a bacteriological laboratory at my command. This viscid acid mucus will not absorb or combine with the antiseptic and germicide lotions of the strength used in the mouth to thoroughly sterilize it. And the tooth brush will not, or any other ordinary means remove it but to a limited extent. I earnestly urge the use of the only harmless agent known that will assist in removing and making it possible to thoroughly cleanse the teeth and gums of this infected deposit. The magnesia hydrate, Mg. H2, O2, has the most fortunate and pleasant action on this viscid acid mucus by neutralizing the acid and precipitating the mag. hyd. in a jelly-like non-clinging mass easily removed by the brush from gums and teeth and carrying away in its mass all germs and spores.

The act of just rinsing the mouth with a small quantity of this mag. hyd. and brushing the teeth places the mouth in a better condition than any mouth antiseptic lotion, and in fact prepares it for the possible therapeutic effect of the lotion that was before mere folly to use.

The 3rd Div. of Stomatocace—Stomatitis Gangrenosa. This distressing condition of the gums and the bony septums of the alveolar process is caused by poisonous medicates. The treatment must be largely systemic. But in all these cases of salivation with purulent discharges through the gums much can be done to relieve the unpleasant condition to the patient and to retard its progress, urging first cleanliness, and the constant use of a germicide lotion to prevent parasitical complications and the direct action of some powerful excarotic astringent to the gums at the places affected. After years of test and careful noting the results, I feel sure that sulphuric acid in about 12 to 20 per cent. applied to the gums down to the process *immediately after the application* of a good syringing out of these pockets and purulent places with hyd. dioxid, surely gives the best result of all. Do not use any application until these spaces between the gums and the teeth are free from pus, and then apply the acid solution on a thin piece of orange wood, I have never discovered the benefits of carbolic acid in a mouth lotion, or in direct treatment of any lesion or disease of the mouth. As to the tinct of myrrh, which is so commonly used, I positively repudiate it, in fact have found it did more injury by leaving an insoluble deposit under the gum border, between the teeth and protected places, and frequently causing gingival irritation or increasing it. Its astringent antiseptic or germicidal properties are far surpassed by other agents. I do not advocate the use of alcoholic tinctures or extracts that lose their integrity, as it were, on coming in contact with the saliva and precipitate a gum or resin. There are many antiseptic lotions presented to us that the manufacturers claim contain such drugs for therapeutical action, such as benzoic acid, myrrh, etc., but when analyzed I fail to find any or but a trace of such drugs, as they are usually left in the filter, having lost their officinal integrity by precipitation on coming in contact with too much water. And again this slight antiseptic action or astringent power amounts to nothing on the pathological conditions found in the mouth that need the most powerful effect to cure. The antiseptic that takes the place of these many popular agents is eucalyptus. Eucalyptol is, I believe, most frequently used, but experience and close observation has caused me to use eucalyptus, and I advise it as the best general antiseptic for a mouth lotion, as its effect is lasting and stable in the mouth, with no injurious qualities.

No. 3. In Calcic Inflammation or Gingivitis—Cleanliness and the removal of all deposits is often all that is needed. If pockets are found wash out with hydrogen dioxid and use general antiseptic lotion given. Stubborn places that will not heal, often caused by the presence of some imbedded parasite, use 12 per cent. tri. chlor. acetic acid or sulphuric acid. Frequently this disease is constitutional and the mucus follicles seem to poison themselves with their own extreme acid excretion. The cause to my mind seems to be fermentations in the large intestines, and good results can be had by giving salol, beechwood creosote, or, best of all, boro glyceride as an intestinal antiseptic and anti-ferment, and continue the treatment until the patient has taken plenty of special out-door physical exercise to give the system the proper tone, more perfect digestion and the intestines the natural movement. The pleasant effect of boric acid and borax on the mucus follicles should not be passed here without a special line to their value. This therapeutical effect is noticed not only in the mouth, but on all excretory small glands and follicles, particularly in the ear, eye, nose and mouth. In acute gingivitis where an antiseptic mouth lotion is indicated, one should be used that contains largely of boric acid, and I prefer one that has a surplus of the floating salt that will lodge around and give more lasting effect.

4th. Phragedemic Pericementitis—or Pyorrhœa Alveolaris, as we all know it, A constitutional symptom positively can be cured by surgical and escarotic antiseptic treatment of the lesion, but it will possibly return to the same part at some future time as long as the systemic causes are present. Treatment, *cleanliness with extra care*, and the use of a good borated mouth wash. Deposits removed by surgical means, pockets and lesion washed out by hyd. dioxid. Then apply to the entire lesion a 12 per cent. tri. chlo. acetic acid.

5th. Intra-Osseous Suppuration—If the abscess or pus is at the apex of the tooth only, treat, if possible, by the excellent antiseptic properties of hyd. dioxid, through the canal, until the pus ceases to form. There is no other treatment or agent that gives the result that this does. If the pus has formed a fistulous track to the gum, force the hyd. dioxid through the canal out if open, until it bubbles out freely on the gum. This can be easily done by filling the cavity with piece of soft dental red rubber force hypodermic needle through into canal and inject. Chronic stubborn cases must be treated from the gum, and by burning off or excising end of root if necrosed, and the fistulous track often has to be washed out with diluted sulphuric acid to assist the expoliation of the dead bone.

6th. Dental Canal and Pulp Antiseptics, and Dental Canal Sepsis—So much has been written on this subject and so many various agents and methods of germicidal and antiseptic treatments are advocated, that I will only advocate that which I believe to be the best, and give the reason, which is thoroughly scientific and true, and why I deem it the best.

In pulp canal antisepsis, before entering or cleansing cavity, put on the rubber dam, *if possible.* Wash out the cavity now with either alcohol or chloroform, as it removes quickest all grease or mucous. Excavate cavity and extract devitalized pulp; enlarge the chamber to its dimensions so as to freely expose the canals, and remove every particle of tissue possible in them. Now apply warm air, not very hot. This must be done slowly, driving out all moisture from canals, and evaporating for some distance the serum in the tubuli, just as it does in a cavity when hot air is used for sensitive dentine. Now close your apical foramen. I am still advocating lead when possible to use it, on account of its germicidal properties as well as its non-irritating qualities, having thoroughly sterilized it before using it, or whatever I use, that it carries in no germs. Up to this time no antiseptic or wash of any order has entered the canal. I now flow the canal full of best quality of oil of cloves, and absorb the surplus with clean and carefully prepared bibulous points that are antiseptically kept bottled for this purpose. I now apply the hot air and vaporize the volatile oil remaining in the canal until apparently dry, then fill the canals, it matters little what, so long as it is free from the germs or spores of micro-organisms.

The oil of cloves seem to give us that success we wish in this delicate antiseptic and germicidal operation that no other antiseptic yet discovered. There are many more powerful antiseptics than the oil of cloves even among the essential oils, but they do not fill the conditions by being as adaptable. The cedrene oils of the the several classes of essential oils seem to give the best results for such conditions. The oil of cloves seems more *compatible to all conditions needed*, and positively gives the success that others do not reach. It is because this oil is absolutely compatible to the serum of the tooth, that it is very diffusible, yet it is so very lasting, and preventing the very first form of fermentation or germ development, and lastly, of a peculiar searching penetration it has when heat is applied to it and it reaches near its volatile degree. Thus, it is driven back into the tubuli and the substance of the tooth embalming it for all eternity, if other conditions hold. It is these cedrene oils that have kept the only organic tissue extent to-day that antedates history, as thus we see in the mummies of ancient Egypt. We have nothing to-day that will embalm and keep organic tissues safe from the destruction of germs for half the time that these cedrene oils have done. The bichloride of mercury that stands the highest of all our modern germicides, fails in time by its continuous chemical action on the tissues it preserves, until it loses its power or destroys what it was to preserve.

The same fortunate result we have in the antiseptic treatment of an exposed pulp. With the above precautions to keep the cavity clean, using no impure water or escarotic in the excavated cavity, but just flow over your *healthy* pulp a few drops of pure oil of cloves and absorb the same with your antiseptic bibulous paper-points, then flow over the pulp and cavity your oxy-phosphate mixed to a creamy constituency and wait until it hardens, putting no pressure on it until it hardens, then balsam over the filling before allowing the saliva to reach it, and you will save more pulps than all the other methods of capping. I do not advocate the use of any escarotic antiseptic on the pulp, it reduces their vitality. The oil of cloves is not an irritant to these tissues, and seems to soothe and completely sterilize it of all germ complications unless they have already made their way into the tissues of the pulp. Be sure you have a healthy pulp at all times, and if in doubt, destroy, in justice to yourself and patient.

In septic canals the treatment as to precaution before entering canal are the same as presented above, but I here advocate drilling out the canals as far as it is expedient at any time to do so. Thus we carry off mechanically much septic tissue and coagulated serum at the mouth of the tubuli that was caused by the fermentation in the canal. I then apply the low heat to drive back the serum; then, if there is no complication outside the foramen, I close it, and apply the oil of cloves to canal, then vaporize with heat, even to passing a hot root dryer into each canal, to vaporize that at the apex and drive it into the tubuli, or any particle of the tissue that may be left in the canal, and embalm it for all eternity. Where there are complications beyond the foramen I treat as suggested in intra-osseous suppuration, and after drill' out the canal to remove the coagulum at the mouth of the tubuli that was caused also by the hyd. dioxid, as there may be some anerobic spores beyond it that the oil of cloves must reach and embalm..

As there are germs all about in all dental offices keep lead points for filling apex and bibulous points for drying out the oil in bottles of powdered boracic acid. The instruments should all be washed in tepid water and caustic soda, then wiped off with a cloth soaked in boro-glyceride, and that is all that is necessary. I do not advocate showing my delicate edge tools in sterilized sand or gravel, or placing them in contact with corrosives.

Thus I close my germicidal and antiseptic treatise, gleaned from a successful practice of eighteen years of close observations and test, always looking for the best.—*Dominion Dental Journal.*

---

### Communicated.

### LIST OF COMMITTEE'S FOR THE JOINT MEETING OF THE SOUTHERN BRANCH OF THE NATIONAL DENTAL ASSOCIATION AND THE LOUISIANA STATE DENTAL SOCIETY.

*To the Editor of "The American Journal of Dental Science."*

